# Impact of CKD on Household Income

**DOI:** 10.1016/j.ekir.2017.12.008

**Published:** 2017-12-23

**Authors:** Rachael L. Morton, Iryna Schlackow, Alastair Gray, Jonathan Emberson, William Herrington, Natalie Staplin, Christina Reith, Kirsten Howard, Martin J. Landray, Alan Cass, Colin Baigent, Borislava Mihaylova, SHARP Collaborative Group, R. Collins, C. Baigent, M.J. Landray, C. Bray, Y. Chen, A. Baxter, A. Young, M. Hill, C. Knott, A. Cass, B. Feldt-Rasmussen, B. Fellström, D.E. Grobbee, C. Grönhagen-Riska, M. Haas, H. Holdaas, L.S. Hooi, L. Jiang, B. Kasiske, U. Krairittichai, A. Levin, Z.A. Massy, V. Tesar, R. Walker, C. Wanner, D.C. Wheeler, A. Wiecek, T. Dasgupta, W. Herrington, D. Lewis, M. Mafham, W. Majoni, C. Reith, J. Emberson, S. Parish, D. Simpson, J. Strony, T. Musliner, L. Agodoa, J. Armitage, Z. Chen, J. Craig, D. de Zeeuw, J.M. Gaziano, R. Grimm, V. Krane, B. Neal, V. Ophascharoensuk, T. Pedersen, P. Sleight, J. Tobert, C. Tomson

**Affiliations:** 1 NHMRC Clinical Trials Centre, The University of Sydney, Sydney, Australia; 2 Health Economics Research Centre, Nuffield Department of Population Health, University of Oxford, UK; 3 Clinical Trial Service Unit and Epidemiological Studies Unit, Nuffield Department of Population Health, University of Oxford, UK; 4 School of Public Health, The University of Sydney, Sydney, Australia; 5 Menzies School of Health Research, Charles Darwin University, Darwin, Australia; 6 Medical Research Council Population Health Research Unit, Nuffield Department of Population Health, University of Oxford, UK

**Keywords:** chronic renal insufficiency, dialysis, income, poverty, transplantation

## Abstract

**Introduction:**

The impact of chronic kidney disease (CKD) on income is unclear. We sought to determine whether CKD severity, serious adverse events, and CKD progression affected household income.

**Methods:**

Analyses were undertaken in a prospective cohort of adults with moderate-to-severe CKD in the Study of Heart and Renal Protection (SHARP), with household income information available at baseline screening and study end. Logistic regressions, adjusted for sociodemographic characteristics, smoking, and prior diseases at baseline, estimated associations during the 5-year follow-up, among (i) baseline CKD severity, (ii) incident nonfatal serious adverse events (vascular or cancer), and (iii) CKD treatment modality (predialysis, dialysis, or transplanted) at study end and the outcome “fall into relative poverty.” This was defined as household income <50% of country median income.

**Results:**

A total of 2914 SHARP participants from 14 countries were included in the main analysis. Of these, 933 (32%) were in relative poverty at screening; of the remaining 1981, 436 (22%) fell into relative poverty by study end. Compared with participants with stage 3 CKD at baseline, the odds of falling into poverty were 51% higher for those with stage 4 (odds ratio [OR]: 1.51; 95% confidence interval [CI]: 1.09–2.10), 66% higher for those with stage 5 (OR: 1.66; 95% CI: 1.11–2.47), and 78% higher for those on dialysis at baseline (OR: 1.78, 95% CI: 1.22–2.60). Participants with kidney transplant at study end had approximately half the risk of those on dialysis or those with CKD stages 3 to 5.

**Conclusion:**

More advanced CKD is associated with increased odds of falling into poverty. Kidney transplantation may have a role in reducing this risk.

Chronic kidney disease (CKD) is a major cause of disability and death worldwide. The incidence of CKD is disproportionately higher among people who are socially disadvantaged^1^, ^2^, ^3^, ^4^ and the disease can have major consequences for the living conditions of individuals and their families, including severe financial problems. Many who progress to dialysis face a potential loss of employment.^5^, ^6^, ^7^ Nonetheless, research on the socioeconomic impact of CKD on households is scarce.

Relative poverty is defined as an income less than 50% of the median income of the country.^8^ It is the state of having insufficient money, goods, or means to maintain a minimum accepted standard of living in a particular society and implies not having enough resources to meet basic needs or access necessary treatment. A recent study described a bidirectional relationship between CKD and poverty,^9^ in which the poor with higher disease burden have fewer resources to meet treatment costs. This results in “catastrophic spending” (defined as out-of-pocket payments exceeding 40% of nonfood expenditure), which further depletes resources and has an impact on entire families. Poverty can also directly affect adherence to medical treatment,^10^ and may result in suboptimal or partial use of medicines to make them last longer.^11^

Although several studies have examined the association between social disadvantage and the incidence of CKD,^1^, ^9^, ^12^, ^13^, ^14^, ^15^ few have examined the relationship between progressive CKD and its impact on household income or poverty. Those who have are limited by retrospective or cross-sectional study designs,^5^, ^13^, ^16^ wherein household income data were collected at 1 single point in time. The aim of this analysis of a large multinational prospective longitudinal study was to determine whether CKD severity and CKD treatment modality were associated with a fall into relative poverty during a median of 5 years of follow-up. We also sought to examine whether common complications in people with CKD, such as myocardial infarction, stroke, and incident cancers affect household income.

## Methods

This prospective study was undertaken in the cohort of participants randomized to simvastatin 20 mg plus ezetimibe 10 mg daily versus matching placebo in the multinational Study of Heart and Renal Protection (SHARP).^17^ SHARP participants were adults aged 40 years or older with moderate-to-severe CKD and no known history of myocardial infarction or coronary revascularization. All participants were screened between June 2003 and June 2006, and final follow-up occurred in 2010.

Participants were included in the current analysis if they reported 2 measures of household income, the first at baseline screening and the second at final study follow-up, approximately 5 years later; referred to hereafter as “screening” and “study end.” To facilitate ease of reporting, participants were presented with 4 household income categories: 2 above and 2 below their country’s median income at the time of screening and again at study end. These were defined as follows: high income (more than twice the country’s median income); medium-high income (more than the median, but less than twice the median); medium-low income (less than the median, but more than half the median); and low income (less than half the median income) (Supplementary Table S1). This final income category is a widely used measure for “relative poverty.” Where possible, the median income levels were obtained from national statistical bureaus. In 4 of 18 countries participating in SHARP, the median income levels used in the study were subsequently found to be inadequately related to the actual median incomes, and participants from these countries were excluded from the analysis. Ethical approval was obtained from all study sites before enrollment.

Participants were followed at screening, and 2 months and 6-monthly thereafter. At each visit, information on all serious adverse events (including vascular events and initiation of renal replacement therapy) was collected; further information was also sought from hospital and other health records. Trained clinicians at the international coordinating center who were masked to study treatment allocation adjudicated major study outcomes using standardized definitions and procedures. Estimated glomerular filtration rates (eGFR) for each participant at each study visit were calculated using the CKD-EPI study equation^18^ and used to establish CKD stage for those not receiving dialysis. At study end, CKD treatment modality was classified as predialysis CKD stage 3 to 5, maintenance dialysis, or kidney transplantation.

To thoroughly examine the impact of progressive CKD on household income, we used 2 “decrease in income category” measures: (i) a fall into relative poverty, defined as a move from a higher income category at screening to the lowest income category (poverty) at study end; and (ii) *any* decrease in income category between screening and study end. (Of note, a change in any household income category represented a major shift in socioeconomic circumstances.) Both “decrease in income category” measures excluded participants who were already in relative poverty at screening.

Explanatory variables included participant age at screening (40–54 years, 55–64 years, 65 years and older); sex; country of recruitment (high income [Australia, Austria, Canada, Czech Republic, Denmark, France, Norway, New Zealand, Poland, United Kingdom, and United States] and middle income [China, Malaysia, and Thailand]); ethnicity (black, white, Asian, or other non-black, dichotomized to black or non-black due to collinearity with the variable “country of recruitment”); number of adults dependent on household income (1, 2 or more, unrecorded); number of children dependent on household income (none, 1 or more, unrecorded); category of household income at screening (high, medium-high, medium-low, low); smoking status (never smoked, former smoker, current smoker); prior vascular disease or prior diabetes; and CKD stage at screening. Allocation to simvastatin plus ezetimibe in the study did not affect progression of CKD^19^ and was not associated with changes in household income in any of the analyses.

### Statistical Methods

Descriptive statistics are presented for participant characteristics by household income category at screening and by measure of household income decrease. The changes in household income category of participants from screening to study end are summarized overall and by CKD stage at screening. The likelihood of a decrease in household income by study end, to the lowest income category (i.e., fall into poverty) was assessed using conditional fixed effects logistic regression, stratified by country. Odds ratios (ORs) with conventional 95% confidence intervals (CIs) are presented throughout the text. All quoted *P* values refer to the comparison with the specified reference category. In tables, to faciliate comparisons among the different categories, group-specific CIs derived from the variance of the logarithm of the OR in each category, including the reference category, are presented when characteristics involve more than 2 categories.^20^, ^21^ The tests for trend across levels of ordered categorical variables (i.e., educational attainment, income, and CKD stages at screening) were calculated using a Wald χ^2^ statistic. All analyses were conducted in STATA v14 (Stata Corp., College Station, TX).

### Sensitivity Analyses

To explore whether serious nonfatal adverse events, such as myocardial infarction, stroke, or incident cancer (excluding nonmelanoma skin cancer), during the follow-up period were associated with a fall into relative poverty, we performed further logisitic regressions including these complications. To explore whether the impact of CKD severity and nonfatal disease events on household income was different in middle-income countries compared with high-income countries, we performed separate analyses in these 2 groups of countries. To investigate whether our results could be affected by participants’ retirement status, and in the absence of retirement data, we performed separate analyses among participants aged younger than 60 years (i.e., potentially employed) and those aged 60 years or older (i.e., likely retired) at study end. To investigate whether the results could differ depending on a participant’s educational attainment and household income at screening, we checked for interactions of effects with these characteristics. In further sensitivity analyses, participants with prior vascular disease or diabetes or of black ethnicity were excluded to investigate robustness of our findings to baseline morbidity. The study is reported according to the STROBE statement for observational studies.^22^

To investigate the relevance of missing income data, 1706 further participants from the 14 study countries with 1 missing income datapoint either at screening or study end were included in sensitivity analysis using multiple imputation with chained equations and 20 imputed sets to impute the missing income category.^23^, ^24^ In addition to income category at screening and study end, covariates used in the multiple imputation model included baseline age, sex, ethnicity, country, education, number of adult and child dependants, smoking status, and prior vascular disease or diabetes; nonfatal adverse events during follow-up; and CKD stage at both baseline and study end. Logistic regression results across imputations were calculated using Rubin’s rules.^23^

## Results

**Figure 1:**
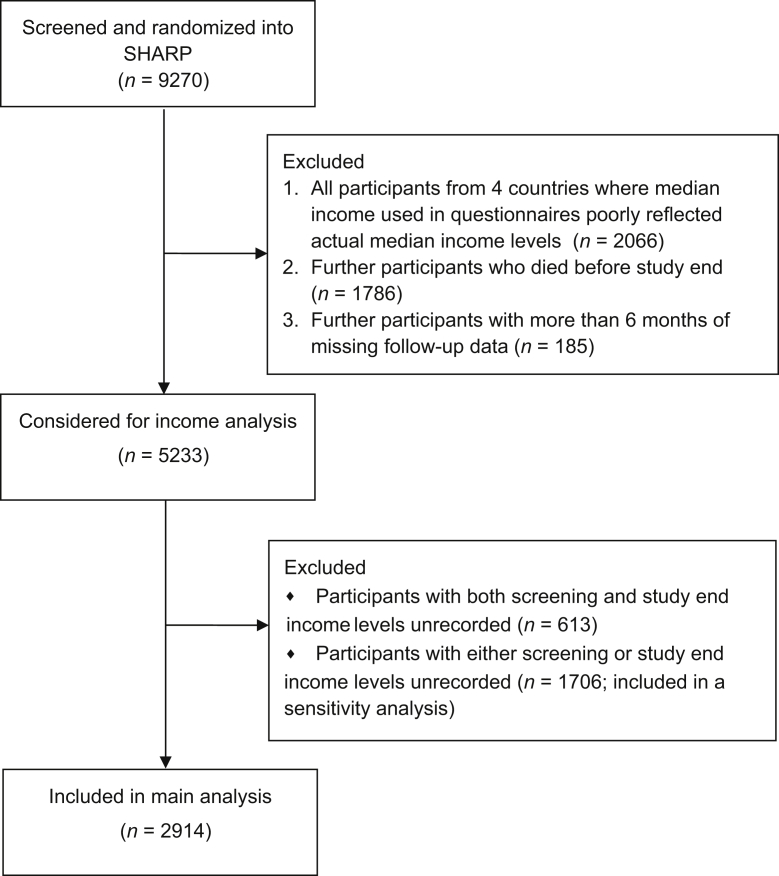
Flowchart of Study of Heart and Renal Protection (SHARP) participants included in household income analysis.

**Figure 2:**
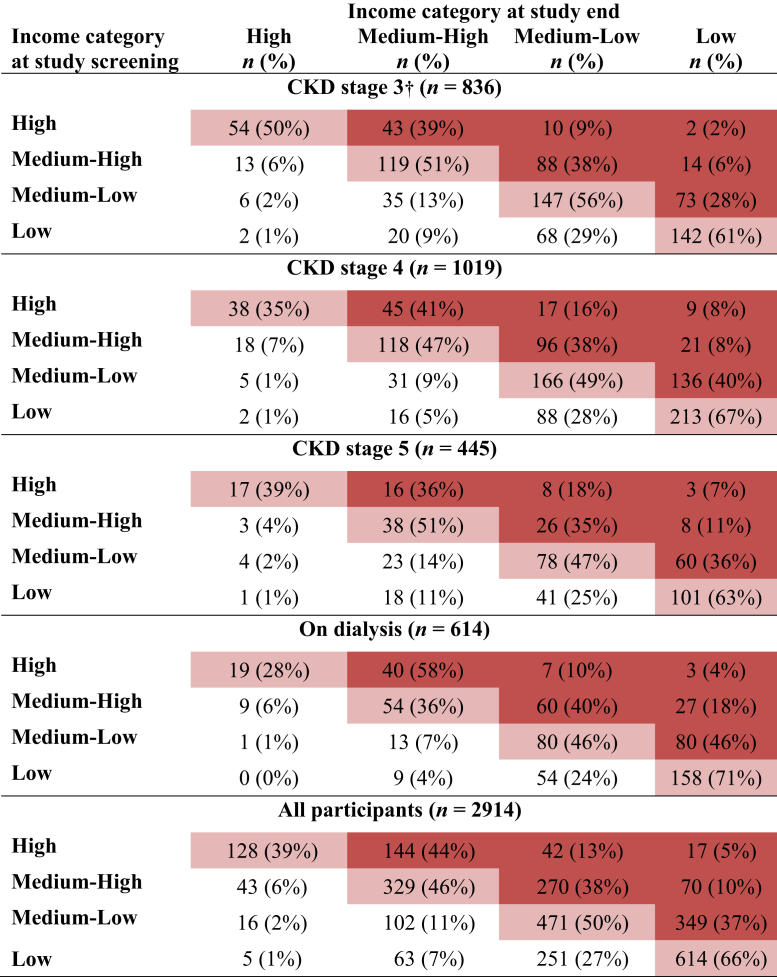
Participants by household income category at screening into Study of Heart and Renal Protection and at study end. By study end, 436 (22%) participants not already in poverty fell into poverty, whereas 892 (45%) moved down at least 1 income category. CKD, chronic kidney disease. The percentages shown are row percentages. †Predominantly CKD stage 3b. Dark red, income category decreased during study; light red, no change; white, income category increased.

A total of 2914 participants from 14 countries, randomized into SHARP and with available income data at screening and study end, were included in the main analysis (Figure 1). An additional 1706 participants with 1 measure of income were included in a sensitivity analysis. The characteristics of the 2914 participants are presented in Supplementary Table S2. Median follow-up for this study cohort was 5.0 years (interquartile range 4.2–5.6); 933 of the 2914 (32%) participants were in the lowest income category for their country (i.e., relative poverty) at screening. This group was more likely to be older, female, of black ethnicity, less educated, have more vascular disease and/or diabetes, and be on dialysis at screening than participants in higher income groups. The baseline characteristics of participants not in poverty at baseline screening who fell into relative poverty, that is, the lowest income category (436 [22%] participants) or into any lower income category (892 [45%] participants) by study end are presented in Table 1. Figure 2 presents the changes in income category for all participants between baseline and study end. Supplementary Table S3 shows CKD status at screening and study end.

**Table 1:** Baseline characteristics of participants not in the lowest income category at screening, by measures of income category decrease

Characteristics	All participants^a^ *n* = 1981	Decrease to lowest income category *n* = 436	Decrease in any income category *n* = 892
Age group (yr)			
40–54	830 (42)	141 (32)	375 (42)
55–64	558 (28)	123 (28)	272 (30)
65 and older	593 (30)	172 (39)	245 (27)
Sex			
Males	1331 (67)	280 (64)	580 (65)
Females	650 (33)	156 (36)	312 (35)
Ethnicity			
White	1459 (74)	344 (79)	705 (79)
Asian (Chinese)	225 (11)	31 (7)	41 (5)
Asian (other)	213 (11)	37 (8)	104 (12)
Black	47 (2)	15 (3)	24 (3)
Other	37 (2)	9 (2)	18 (2)
Highest education level			
Tertiary	471 (24)	43 (10)	195 (22)
Completed high school	392 (20)	79 (18)	163 (18)
Vocational qualifications	436 (22)	112 (26)	201 (23)
Completed lower high school	408 (21)	113 (26)	196 (22)
Completed primary school	234 (12)	79 (18)	119 (13)
No formal education	33 (2)	10 (2)	17 (2)
Unrecorded	7 (0)	0 (0)	1 (0)
Income category			
High	331 (17)	17 (4)	203 (23)
Medium-High	712 (36)	70 (16)	340 (38)
Medium-Low	938 (47)	349 (80)	349 (39)
Low	-	-	-
Number of child dependants in household			
None	1384 (70)	349 (80)	640 (72)
One or more	521 (26)	75 (17)	223 (25)
Unrecorded	76 (4)	12 (3)	29 (3)
Number of adult dependants in household			
One	376 (19)	125 (29)	169 (81)
Two or more	1597 (81)	309 (71)	720 (19)
Unrecorded	8 (0)	2 (0)	3 (0)
Smoking status			
Never	1052 (53)	206 (47)	454 (51)
Former	731 (37)	182 (42)	345 (39)
Current	198 (10)	48 (11)	93 (10)
Prior diseases			
Vascular disease	190 (10)	52 (12)	92 (10)
Diabetes	310 (16)	76 (17)	138 (15)
CKD stage			
CKD 3^b^	604 (30)	89 (20)	230 (26)
CKD 4	700 (35)	166 (38)	324 (36)
CKD 5	284 (14)	71 (16)	121 (14)
On dialysis	393 (20)	110 (25)	217 (24)
Follow-up years, mean (SD)	4.98 (0.72)	5.02 (0.73)	5.03 (0.72)

CKD, chronic kidney disease.

Values are *n* (%) unless otherwise indicated.

a Participants’ data by measures of decrease in income category excludes the 933 participants who were in the lowest income category at screening. Column percentages are presented.

b Predominantly CKD stage 3b.

After adjustment for socioeconomic predictors of poverty, including black ethnicity, low educational attainment, a single-adult household, and income category at screening, CKD severity at screening was strongly associated with a fall into relative poverty by study end (Table 2). Compared with participants with stage 3 CKD at baseline, the odds of falling into relative poverty were 51% higher for those with stage 4 CKD (OR: 1.51; 95% CI: 1.09–2.10), 66% higher for those with stage 5 CKD (OR: 1.66; 95% CI: 1.11–2.47), and 78% higher for those on dialysis at baseline (OR: 1.78; 95% CI: 1.22–2.60) (Table 2). These findings were not affected by the exclusion of participants of black ethnicity or with vascular disease or diabetes at baseline.

**Table 2:** Factors associated with the likelihood of a fall into poverty, a multivariate logistic regression

Characteristics at screening	OR (Conventional 95% CI)	(Group-specific 95% CI)
Age group (yr)		
40–54	1.0	(0.77–1.29)
55–64	1.19 (0.85–1.66)	(0.96–1.47)
65 and older	1.17 (0.83–1.65)	(0.93–1.47)
Sex		
Males (vs. females)	0.92 (0.70–1.20)	—
Ethnicity		
Black (vs. non-black)	3.37 (1.40–8.14)	—
Highest educational attainment		
Tertiary	1.0	(0.70–1.43)
Completed high school	1.63 (1.04–2.57)	(1.20–2.22)
Vocational qualifications	2.09 (1.36–3.21)	(1.63–2.69)
Completed lower high school	2.25 (1.46–3.48)	(1.76–2.89)
Completed primary school	2.66 (1.64–4.30)	(1.93–3.66)
No formal education	1.71 (0.70–4.18)	(0.76–3.86)
Baseline income		
High	1.0	(0.60–1.68)
Medium-high	2.00 (1.13–3.53)	(1.57–2.54)
Medium-low	10.50 (6.03–18.29)	(8.58–12.84)
Number of adult dependants		
Two or more	1.0	(0.83–1.20)
One	1.67 (1.24–2.25)	(1.31–2.12)
Unrecorded	3.28 (0.56–19.38)	(0.56–19.20)
Number of child dependants		
One or more	1.0	(0.73–1.38)
None	1.23 (0.86–1.76)	(1.04–1.45)
Unrecorded	0.88 (0.41–1.91)	(0.43–1.78)
Smoking status		
Never smoked	1.0	(0.83–1.20)
Prior smoker	1.23 (0.94–1.62)	(1.01–1.51)
Current smoker	1.55 (1.00–2.38)	(1.05–2.29)
Prior diseases		
Vascular disease	1.34 (0.90–1.99)	—
Diabetes mellitus	1.07 (0.76–1.50)	—
CKD stage		
CKD 3^a^	1.0	(0.78–1.29)
CKD 4	1.51 (1.09–2.10)	(1.24–1.86)
CKD 5	1.66 (1.11–2.47)	(1.22–2.26)
On dialysis	1.78 (1.22–2.60)	(1.35–2.36)

CI, confidence interval; CKD, chronic kidney disease; OR, odds ratio.

The logistic regression model was further stratified by country. Wald χ^2^ test for trend across CKD stages, χ^2^ = 9.19, *P* = 0.0024. The dashes indicate binary characteristics (e.g., males/females) in which group-specific 95% CI are not relevant.

a Predominantly CKD stage 3b.

The further inclusion of CKD stage at study end (CKD stage 3–5 not on dialysis [*n* = 1065], dialysis [*n* = 571], or kidney transplant [*n* = 345]) into the multivariate logistic model showed that, compared with participants in CKD stage 3 to 5 at study end, the odds of falling into relative poverty were 55% lower for those who received a kidney transplant (occurring an average of 2.5 years before study end; OR: 0.45; 95% CI: 0.27–0.74) but were not significantly changed by further adjustment for those on dialysis at study end (OR: 0.92; 95% CI: 0.64–1.32; Table 3). Further split of participants with kidney transplant into preemptive transplant (i.e., without a preceeding period of dialysis; *n* = 64 participants) or non-preemptive transplant (*n* = 281) showed that both were associated with a similarly reduced likelihood of poverty (OR: 0.17; 95% CI: 0.04–0.75, and OR: 0.52; 95% CI 0.31–0.87, respectively, compared with CKD stage 3 to 5 at study end). There were no important differences in effects by participants’ CKD status, educational attainment, or household income at screening.

**Table 3:** Contribution of nonfatal vascular events, incident cancers, and CKD severity at study end to the likelihood of a fall into poverty

Category of event	OR (Conventional 95% CI)	(Group-specific 95% CI)
Nonfatal myocardial infarction (*n* = 48)	1.84 (0.89–3.84)	-
Nonfatal stroke (*n* = 44)	0.86 (0.39–1.88)	-
Incident cancer (*n* = 187)	1.05 (0.70–1.57)	-
Composite^a^ of nonfatal events (*n* = 264)	1.12 (0.79–1.59)	-
CKD status at study end		
CKD stage 3–5 (*n* = 1065)	1.0	(0.73–1.37)
Transplant^b^ (*n* = 345)	0.45 (0.27–0.74)	(0.31–0.66)
Dialysis (*n* = 571)	0.92 (0.64–1.32)	(0.77–1.09)

Multivariate logistic regression models stratified by country and adjusted for age, sex, black ethnicity, education level, number of adult and child dependants, baseline income, smoking, prior vascular disease, prior diabetes and CKD stage at screening.

a Composite of nonfatal mycocardial infarctions, strokes, and incident cancers.

b Transplant at study end split by type of transplant: preemptive transplant (*n* = 64) OR: 0.17 (Conventional 95% CI: 0.04–0.75) and non-preemptive transplant (*n* = 281) OR: 0.52 (0.31–0.87) in fully adjusted multivariate model, not significantly different χ^2^ = 2.05, *P* = 0.1521.

In sensitivity analyses, serious nonfatal cardiovascular events or cancers during the study (occurring an average of 2.5 years before study end), separately or as a composite, were not associated with the likelihood of falling into poverty (Table 3). Results of the analyses for the outcome of *any* decrease in household income category, excluding participants who were in poverty at screening, were similar to results for a fall into relative poverty. Increased severity of CKD was associated with an increased likelihood of decrease in income category (Supplementary Table S4). Compared with participants in CKD stage 3 to 5 at study end, the odds of decreasing household income category were lower for those who received a kidney transplant (OR: 0.63; 95% CI: 0.44–0.89), and similar for those on dialysis at study end (OR: 1.23; 95% CI: 0.92–1.64) (Supplementary Table S5). Again, nonfatal myocardial infarction, stroke, or incident cancer were not associated with decrease in any income category.

A greater proportion of study participants in middle- compared with high-income countries were in relative poverty at baseline screening (52% [433/830] vs. 24% [500/2084]), but the associations between CKD severity at baseline and a fall into poverty were similar for high- and middle-income countries (Supplementary Table S6). The associations between CKD severity at baseline and fall into relative poverty were also similar for younger participants, aged <60 years at study end, and for older participants aged ≥60 years at study end (Supplementary Table S7). The results of sensitivity analyses including participants with 1 missing income endpoint (more likely to be female, of black ethnicity, and on dialysis at screening but similar with respect to other characteristics) and following multiple imputation were consistent with the main analyses (Supplementary Table S8).

## Discussion

Our results show that, in addition to the known social determinants of poverty, such as low education, living alone, and black ethnicity, more advanced CKD is associated with increased odds of falling into relative poverty. Participants in receipt of a kidney transplant, however, had lower risk of poverty compared with those in other CKD stages at study end following adjustments for demographic, socioeconomic, and disease characteristics. By contrast, we found no evidence that experiencing nonfatal events, such as myocardial infarction, stroke, or incident cancer, during the follow-up period influenced household income.

Several factors might drive the association between kidney transplantation and a reduced likelihood of falling into relative poverty. First, successful kidney transplantation has been shown to be associated with return to work, with studies reporting that approximately 60% of transplanted patients remain in or return to paid employment 2 years after receiving a transplant.^25^, ^26^ Second, although the cost of treatment for CKD borne by patients varies between countries,^12^ ongoing costs related to transplantation are substantially lower than those for maintenance dialysis.^27^ Nevertheless, it is possible that unmeasured confounders related to low socioeconomic status, such as less knowledge about transplantation and limited engagement in health care decision-making^28^ may have contributed to this association. In addition, a receipt of a kidney transplant can indicate better health status (all else being equal), and despite our efforts to account for a wide range of circumstances, some bias may remain. Therefore, this novel finding should be considered hypothesis generating and confirmed in further longitudinal studies. In particular, further research is needed to assess the productivity gains associated with transplantation at individual, household, and broader population levels.

A previous cross-sectional study of 625 German participants managed on chronic hemodialysis^16^ reported that younger age (younger than 50 years) and having more than 2 adults living in the household were associated with a greater likelihood of poverty. We found no evidence of this; in fact, in our study, adults who lived alone were significantly more likely to fall into poverty. The nonsignificant association between nonfatal adverse events and changes in household income in the present study is likely to be influenced by several factors. First, nearly half of participants experiencing myocardial infarction, stroke, or incident cancer during follow-up died before study end and could not be included in this analysis; therefore, the nonfatal events studied may represent a selection of less severe events. Second, the relatively small number of such nonfatal events may be insufficient to estimate reliably any association. Finally, rehabilitation from these possibly less severe nonfatal myocardial infarctions and strokes may be achieved in a few months. Thus, the loss of income may be negligible if disruption to an individual’s employment is temporary, if individuals are covered by sick leave or health insurance, or if the household income is supplemented by other working adults.^29^, ^30^

There are some limitations in our study to acknowledge. First, we did not have complete income data at screening and study end for all surviving SHARP participants. However, the results of sensitivity analyses including all 4620 participants with at least 1 income measurement and imputing missing data, using the sociodemographic and health information available, were consistent with the main study findings. Second, we did not have data about participants’ employment status or health insurance status, factors likely to influence household income and possibly further related to the likelihood of receiving a kidney transplant, and instead used broad sensitivity analyses to check sensitivity of our findings. Third, SHARP participants from 4 of 18 participating countries had to be excluded from our analysis due to income categorization at screening or study end that turned out to inadequately reflect actual median income levels. Fourth, the inclusion criteria into SHARP meant that study participants, although with advanced CKD, were with no known history of myocardial infarction or coronary revascularization and, therefore, were healthier than average patients with CKD in the community. The impact of advanced CKD on household income of patients with more morbidities, however, is likely to be even larger.

This large study suggests that a substantial proportion of adults with moderate-to-severe CKD are living in relative poverty, and many more will fall into poverty as their CKD progresses. An implication of these findings is that advanced CKD should be considered as a poverty risk factor alongside other serious chronic diseases and social determinants of poverty such as low education level, employment status, and ethnicity. Kidney transplantation for those with more advanced CKD may have a role in reducing the risk of household poverty.

## Disclosure

All the authors declared no competing interests.

## Supplementary Material

Table S1 Median household income per year used to define questionnaire categories in the SHARP study for 14 participating countries at screening and study end.

Table S2 Baseline characteristics of the 2914 participants with income category data available at screening and study end.

Table S3 CKD status at screening and study end.

Table S4 Factors associated with the likelihood of a decrease in income category, multivariate logistic regression.

Table S5 Contribution of nonfatal vascular events, incident cancers, and CKD severity at study end to the likelihood of a decrease in any income category.

Table S6 Factors associated with the likelihood of fall into poverty, by high or low-middle income country category.

Table S7 Factors associated with the likelihood of fall into poverty, by participants’ age at study end.

Table S8 Factors associated with the likelihood of a fall into poverty, multivariate logistic regressions following multiple imputation of missing income data.
