# Supplementary material for: Impact of CKD on Household Income
Source: Kidney Int Rep. 2017 Dec 23;3(3):610–8. doi: 10.1016/j.ekir.2017.12.008 (PMC5976816; doi:10.1016/j.ekir.2017.12.008)
Supplement: Table S1 — Median household income per year used to define questionnaire categories in the SHARP study for 14 participating countries at screening and study end. [file mmc1.docx]

**Table S1. Median household income per year used to define questionnaire categories in the SHARP study for 14 participating countries at screening and study end**

| **Country*** | **Number of participants** | **Country median household income at study screening** | **Country median household income at study end** |
| --- | --- | --- | --- |
| **High-income countries** | | | |
| Australia | 367 | AUD $28,000 | AUD $53,000 |
| Austria | 15 | EUR €23,900 | EUR €28,000 |
| Canada | 206 | CAD $46,800 | CAD $54,000 |
| Czech Republic | 54 | CZK Kč120,000 | CZK Kč354,000 |
| Denmark | 84 | DKK kr417,500 | DKK kr500,000 |
| France | 58 | EUR €29,200 | EUR €36,000 |
| Norway | 84 | NOK kr409,000 | NOK kr576,000 |
| New Zealand | 153 | NZD $40,600 | NZD $58,000 |
| Poland | 49 | PLN zł25,100 | PLN zł33,000 |
| United Kingdom | 879 | GBP £22,000 | GBP £28,000 |
| United States | 135 | USD $42,000 | USD $51,000 |
| **Middle-income countries** | | | |
| China | 504 | CNY ¥24,200 | CNY ¥43,000 |
| Malaysia | 204 | MYR RM37,200 | MYR RM44,000 |
| Thailand | 122 | THB ฿161,000 | THB ฿224,000 |

*Data from participants in Germany, Sweden, Finland and Netherlands excluded as the median income data presented in patient study questionnaires poorly reflected actual income levels in that country.

Median household income data sourced from:

Australian Bureau of Statistics <http://www.abs.gov.au/census>

Statistics Austria <http://www.statistik.at/web_en/>

Statistics Canada <http://www.statcan.gc.ca/start-debut-eng.html>

National Bureau of Statistics of China <http://www.stats.gov.cn/english/>

Czech Statistical Office <https://www.czso.cz/csu/czso/home>

Statistics Denmark <http://www.dst.dk/en>

National Institute of Statistics and Economic Studies <http://www.insee.fr/en/>

Department of Statistics Malaysia <http://www.statistics.gov.my/>

Statistics Norway <http://www.ssb.no/>

Statistics New Zealand <http://www.stats.govt.nz/>

Central Statistical Office of Poland <http://stat.gov.pl/en/>

National Statistical Office of Thailand <http://web.nso.go.th/>

Office for National Statistics <http://www.ons.gov.uk/ons/index.html>

United States Census Bureau <http://www.census.gov/>
