# Supplementary material for: Impact of CKD on Household Income
Source: Kidney Int Rep. 2017 Dec 23;3(3):610–8. doi: 10.1016/j.ekir.2017.12.008 (PMC5976816; doi:10.1016/j.ekir.2017.12.008)
Supplement: Table S2 — Baseline characteristics of the 2914 participants with income category data available at screening and study end. [file mmc2.docx]

**Table S2. Baseline characteristics of the 2914 participants with income category data available at screening and study end**

|  | **Participants in the ‘low’ income (ie, relative poverty) category at screening**  **n=933** | **Other participants**  **n=1981** | **All participants**  **n=2914** |
| --- | --- | --- | --- |
| *Age group (years)* |  |  |  |
| 40-54 | 332 (36%) | 830 (42%) | 1162 (40%) |
| 55-64 | 251 (27%) | 558 (28%) | 809 (28%) |
| 65 and older | 350 (38%) | 593 (30%) | 943 (32%) |
|  |  |  |  |
| *Sex* |  |  |  |
| Males | 524 (56%) | 1331 (67%) | 1855 (64%) |
| Females | 409 (44%) | 650 (33%) | 1059 (36%) |
|  |  |  |  |
| *Ethnicity* |  |  |  |
| White | 415 (44%) | 1459 (74%) | 1874 (64%) |
| Asian (Chinese) | 279 (30%) | 225 (11%) | 504 (17%) |
| Asian (Other) | 172 (18%) | 213 (11%) | 385 (13%) |
| Black | 43 (5%) | 47 (2%) | 90 (3%) |
| Other | 24 (3%) | 37 (2%) | 61 (2%) |
|  |  |  |  |
| *Highest education level* |  |  |  |
| Tertiary | 54 (6%) | 471 (24%) | 525 (18%) |
| Completed high school | 140 (15%) | 392 (20%) | 532 (18%) |
| Vocational qualifications | 163 (17%) | 436 (22%) | 599 (21%) |
| Completed lower high school | 259 (28%) | 408 (21%) | 667 (23%) |
| Completed primary school | 253 (27%) | 234 (12%) | 487 (17%) |
| No formal education | 55 (6%) | 33 (2%) | 88 (3%) |
| Unrecorded | 9 (1%) | 7 (0%) | 16 (1%) |
|  |  |  |  |
| *Income category* |  |  |  |
| High | - | 331 (17%) | 331 (11%) |
| Medium-High | - | 712 (36%) | 712 (24%) |
| Medium-Low | - | 938 (47%) | 938 (32%) |
| Low | 933 (100%) | - | 933 (32%) |
|  |  |  |  |
| *Number of child dependants in household* |  |  |  |
| None | 630 (68%) | 1384 (70%) | 2014 (69%) |
| One or more | 230 (25%) | 521 (26%) | 751 (26%) |
| Unrecorded | 73 (8%) | 76 (4%) | 149 (5%) |
|  |  |  |  |
| *Number of adult dependants in household* |  |  |  |
| One | 412 (44%) | 376 (19%) | 788 (27%) |
| Two or more | 463 (50%) | 1597 (81%) | 2060 (71%) |
| Unrecorded | 58 (6%) | 8 (0%) | 66 (2%) |
|  |  |  |  |
| *Smoking status* |  |  |  |
| Never | 543 (58%) | 1052 (53%) | 1595 (55%) |
| Former | 260 (28%) | 731 (37%) | 991 (34%) |
| Current | 130 (14%) | 198 (10%) | 328 (11%) |
|  |  |  |  |
| *Prior diseases* |  |  |  |
| Vascular disease | 127 (14%) | 190 (10%) | 317 (11%) |
| Diabetes | 223 (24%) | 310 (16%) | 533 (18%) |
|  |  |  |  |
| *CKD stage* |  |  |  |
| CKD 3^†^ | 232 (25%) | 604 (30%) | 836 (29%) |
| CKD 4 | 319 (34%) | 700 (35%) | 1019 (35%) |
| CKD 5 | 161 (17%) | 284 (14%) | 445 (15%) |
| On dialysis | 221 (24%) | 393 (20%) | 614 (21%) |
|  |  |  |  |
| Follow-up years, mean (SD) | 4.92 (0.76) | 4.98 (0.72) | 4.96 (0.74) |

CKD, chronic kidney disease. SD, standard deviation.

Column percentages are presented.

†Predominantly CKD stage 3b
