# Supplementary material for: Impact of CKD on Household Income
Source: Kidney Int Rep. 2017 Dec 23;3(3):610–8. doi: 10.1016/j.ekir.2017.12.008 (PMC5976816; doi:10.1016/j.ekir.2017.12.008)
Supplement: Table S3 — CKD status at screening and study end. [file mmc3.docx]

**Table S3. CKD status at screening and study end**

| **CKD stage at screening** |  | **CKD stage at study end** | | | | | |
| --- | --- | --- | --- | --- | --- | --- | --- |
|  |  | **3** | **4** | **5** | **On dialysis** | **Transplanted** | **Total** |
|  | **3** | 460 | 257 | 51 | 54 | 14 | **836** |
|  | **4** | 96 | 417 | 162 | 235 | 109 | **1019** |
|  | **5** | 4 | 21 | 72 | 259 | 89 | **445** |
|  | **On dialysis** | 2 | 4 | 3 | 391 | 214 | **614** |
|  | **Transplanted** | 0 | 0 | 0 | 0 | 0 | **0** |
|  | **Total** | **562** | **699** | **288** | **939** | **426** | **2914** |

CKD, chronic kidney disease.
