# Supplementary material for: Impact of CKD on Household Income
Source: Kidney Int Rep. 2017 Dec 23;3(3):610–8. doi: 10.1016/j.ekir.2017.12.008 (PMC5976816; doi:10.1016/j.ekir.2017.12.008)
Supplement: Table S4 — Factors associated with the likelihood of a decrease in income category, multivariate logistic regression. [file mmc4.docx]

**Table S4. Factors associated with the likelihood of a decrease in income category, multivariate logistic regression**

| **Characteristics at screening** | **OR**  **(Conventional 95% CI)** | **(Group-specific 95% CI)** |
| --- | --- | --- |
| *Age group (years)* |  |  |
| 40-54 | 1.0 | (0.82-1.21) |
| 55-64 | 1.34 (1.04-1.73) | (1.13-1.58) |
| 65 and older | 1.07 (0.81-1.42) | (0.88-1.31) |
|  |  |  |
| *Sex* |  |  |
| Males *(vs Females)* | 0.80 (0.64-0.99) | - |
|  |  |  |
| *Ethnicity* |  |  |
| Black *(vs non-Black)* | 2.32 (1.14-4.72) | - |
|  |  |  |
| *Highest educational attainment* |  |  |
| Tertiary | 1.0 | (0.80-1.25) |
| Completed high school | 1.18 (0.87-1.62) | (0.94-1.49) |
| Vocational qualifications | 1.61 (1.19-2.17) | (1.31-1.97) |
| Completed lower high school | 1.65 (1.21-2.25) | (1.34-2.04) |
| Completed primary school | 1.89 (1.31-2.74) | (1.42-2.53) |
| No formal education | 1.91 (0.90-4.05) | (0.93-3.91) |
| Unrecorded | 0.18 (0.02-1.80) | (0.02-1.78) |
|  |  |  |
| *Baseline income* |  |  |
| High | 1.0 | (0.77-1.30) |
| Med-high | 0.53 (0.39-0.71) | (0.46-0.61) |
| Med-low | 0.29 (0.21-0.39) | (0.24-0.34) |
|  |  |  |
| *Number of adult dependants* |  |  |
| Two or more | 1.0 | (0.87-1.15) |
| One | 1.29 (0.99-1.67) | (1.03-1.61) |
| Unrecorded | 1.17 (0.19-7.11) | (0.20-7.07) |
|  |  |  |
| *Number of child dependants* |  |  |
| One or more | 1.0 | (0.80-1.25) |
| None | 1.24 (0.96-1.61) | (1.09-1.41) |
| Unrecorded | 0.88 (0.50-1.54) | (0.53-1.47) |
|  |  |  |
| *Smoking status* |  |  |
| Never smoked | 1.0 | (0.87-1.15) |
| Prior smoker | 1.16 (0.93-1.43) | (0.98-1.36) |
| Current smoker | 1.28 (0.90-1.82) | (0.93-1.77) |
|  |  |  |
| *Prior diseases* |  |  |
| Vascular disease | 1.33 (0.95-1.86) | - |
| Diabetes mellitus | 1.05 (0.79-1.39) | - |
|  |  |  |
| *CKD stage* |  |  |
| CKD 3† | 1.0 | (0.84-1.20) |
| CKD 4 | 1.29 (1.01-1.64) | (1.10-1.52) |
| CKD 5 | 1.27 (0.93-1.75) | (0.98-1.65) |
| Dialysis | 1.49 (1.12-1.98) | (1.19-1.87) |

CKD, chronic kidney disease. OR, odds ratio. CI, confidence interval.

The logistic regression model was further stratified by country.

†Predominantly CKD stage 3b

Wald chi-square test for trend across CKD stages, χ^2^=6.88, p=0.0087
