# Supplementary material for: Impact of CKD on Household Income
Source: Kidney Int Rep. 2017 Dec 23;3(3):610–8. doi: 10.1016/j.ekir.2017.12.008 (PMC5976816; doi:10.1016/j.ekir.2017.12.008)
Supplement: Table S5 — Contribution of nonfatal vascular events, incident cancers, and CKD severity at study end to the likelihood of a decrease in any income category. [file mmc5.docx]

**Table S5. Contribution of nonfatal vascular events, incident cancers and CKD severity at study end to the likelihood of a decrease in *any income category***

| **Category of event** | **OR**  **(Conventional 95% CI)** | **(Group-specific 95% CI)** |
| --- | --- | --- |
| *Nonfatal myocardial infarction (n=48)* | 1.61 (0.86-3.02) | - |
| *Nonfatal stroke (n=44)* | 0.77 (0.39-1.52) | - |
| *Incident cancer (n=187)* | 1.09 (0.79-1.53) | - |
| *Composite* of nonfatal events (n=264)* | 1.10 (0.82-1.46) | - |
|  |  |  |
| *CKD status at study end* |  |  |
| CKD stage 3-5 (n=1065) | 1.0 | 1.0 (0.79-1.27) |
| Transplant^†^ (n=345) | 0.63 (0.44-0.89) | 0.63 (0.48-0.82) |
| Dialysis (n=571) | 1.23 (0.92-1.64) | 1.23 (1.04-1.45) |

CKD, chronic kidney disease. OR, odds ratio. CI, confidence interval.

Multivariate logistic regression models stratified by country and adjusted for age, sex, black ethnicity, education level, number of adult and child dependants, baseline income, smoking, prior vascular disease, prior diabetes and CKD stage at screening.

^†^Transplant at study end split by type of transplant: preemptive transplant (n=64) OR 0.39 (Conventional 95% CI: 0.21 -0.73) and non-preemptive transplant (n=281) OR 0.73 (0.49-1.07) in fully adjusted multivariate model, not significantly different χ^2^=3.35, p=0.0673.

*Composite of nonfatal myocardial infarctions, strokes, and incident cancers
