# Supplementary material for: Impact of CKD on Household Income
Source: Kidney Int Rep. 2017 Dec 23;3(3):610–8. doi: 10.1016/j.ekir.2017.12.008 (PMC5976816; doi:10.1016/j.ekir.2017.12.008)
Supplement: Table S6 — Factors associated with the likelihood of fall into poverty, by high or low-middle income country category. [file mmc6.docx]

**Table S6. Factors associated with the likelihood of fall into poverty, by high or low-middle income country category**

| **Participant characteristic at screening** | **High-Income Countries (n=1,584)** | | **Middle-Income Countries (n=397: China n=225, Malaysia n=64, Thailand n=108)** | |
| --- | --- | --- | --- | --- |
|  | **OR**  **(Conventional 95% CI)** | **(Group-specific 95% CI)** | **OR**  **(Conventional 95% CI)** | **(Group-specific 95% CI)** |
| *Age group (years)* |  |  |  |  |
| 40-54 | 1.0 | (0.75-1.34) | 1.0 | (0.54-1.84) |
| 55-64 | 1.07 (0.74-1.56) | (0.85-1.36) | 2.91 (1.28-6.64) | (1.67-5.08) |
| 65 and older | 1.24 (0.84-1.82) | (0.96-1.59) | 1.06 (0.42-2.68) | (0.53-2.14) |
|  |  |  |  |  |
| *Sex* |  |  |  |  |
| Males *(vs Females)* | 0.97 (0.71-1.31) | - | 0.59 (0.27-1.31) | - |
|  |  |  |  |  |
| *Ethnicity** |  |  |  |  |
| Black *(vs non-Black)* | 3.32 (1.37-8.06) | - | - | - |
|  |  |  |  |  |
| *Highest educational attainment* |  |  |  |  |
| Tertiary | 1.0 | (0.69-1.45) | 1.0 | (0.36-2.75) |
| Completed high school | 1.65 (0.99-2.73) | (1.17-2.33) | 1.59 (0.50-5.04) | (0.98-2.58) |
| Vocational qualifications | 2.07 (1.30-3.30) | (1.62-2.65) | 2.66 (0.78-9.16) | (1.28-5.53) |
| Completed lower high school | 2.30 (1.43-3.70) | (1.79-2.95) | 2.59 (0.79-8.45) | (1.40-4.79) |
| Completed primary school | 3.19 (1.88-5.43) | (2.27-4.50) | 1.10 (0.28-4.25) | (0.51-2.36) |
| No formal education | 1.87 (0.73-4.75) | (0.81-4.32) | § | § |
|  |  |  |  |  |
| *Baseline income* |  |  |  |  |
| High | 1.00 | (0.59-1.69) | reference† | reference† |
| *(Medium-high)* | 1.63 (0.91-2.93) | (1.25-2.12) | - | - |
| Medium-low | 9.41 (5.31-16.66) | (7.49-11.81) | 3.14 (1.38-7.15) | - |
|  |  |  |  |  |
| *Number of adult dependants* |  |  |  |  |
| Two or more | 1.0 | (0.82-1.22) | 1.0 | - |
| One | 2.01 (1.44-2.81) | (1.54-2.63) | 0.82 (0.37-1.82) | - |
| Unrecorded | 7.39 (0.88-61.88) | (0.89-61.28) | § | § |
|  |  |  |  |  |
| *Number of child dependants* |  |  |  |  |
| One or more | 1.0 | (0.67-1.49) | 1.0 | (0.57-1.77) |
| None | 1.28 (0.84-1.95) | (1.11-1.48) | 0.91 (0.42-1.98) | (0.54-1.55) |
| Unrecorded | 0.87 (0.34-2.21) | (0.37-2.02) | 0.86 (0.18-4.12) | (0.20-3.70) |
|  |  |  |  |  |
| *Smoking status* |  |  |  |  |
| Never smoked | 1.0 | (0.82-1.22) | 1.0 | (0.53-1.89) |
| Prior smoker | 1.17 (0.88-1.56) | (0.95-1.44) | 2.42 (0.91-6.40) | (1.16-5.05) |
| Current smoker | 1.48 (0.92-2.40) | (0.96-2.29) | 2.84 (0.89-9.08) | (1.07-7.52) |
|  |  |  |  |  |
| *Prior diseases* |  |  |  |  |
| Vascular disease | 1.52 (0.98-2.35) | - | 0.68 (0.21-2.22) | - |
| Diabetes mellitus | 1.28 (0.87-1.90) | - | 0.52 (0.24-1.15) | - |
|  |  |  |  |  |
| *CKD stage* |  |  |  |  |
| CKD 3 | 1.0 | (0.76-1.32) | 1.0 | (0.50-1.99) |
| CKD 4 | 1.42 (1.00-2.04) | (1.14-1.78) | 2.24 (0.91-5.50) | (1.26-3.97) |
| CKD 5 | 1.30 (0.82-2.06) | (0.90-1.87) | 3.51 (1.40-8.80) | (1.88-6.56) |
| Dialysis | 1.68 (1.12-2.51) | (1.25-2.25) | 2.39 (0.79-7.29) | (1.00-5.71) |

CKD, chronic kidney disease. OR, odds ratio. CI, confidence interval.

*No participants of black ethnicity in middle-income countries

§ Not estimated due to small numbers

†Participants in high and medium-high income groups were combined due to small number participants in high-income group.

Wald chi-square test for trend across CKD stages: High-income countries χ^2^=5.01, p=0.0251; Middle-income countries χ^2^
