# Supplementary material for: Impact of CKD on Household Income
Source: Kidney Int Rep. 2017 Dec 23;3(3):610–8. doi: 10.1016/j.ekir.2017.12.008 (PMC5976816; doi:10.1016/j.ekir.2017.12.008)
Supplement: Table S7 — Factors associated with the likelihood of fall into poverty, by participants’ age at study end. [file mmc7.docx]

**Table S7. Factors associated with the likelihood of fall into poverty, by participants’ age at study end**

| **Characteristics at screening** | **<60 years (n=809)** | | | | **≥60 years (n=1168†)** | | | |  |
| --- | --- | --- | --- | --- | --- | --- | --- | --- | --- |
|  | **OR**  **(Conventional 95% CI)** | | **(Group-specific 95% CI)** | | **OR**  **(Conventional 95% CI)** | | **(Group-specific 95% CI)** | |  |
| *Sex* | |  | |  | |  | |  | |
| Males *(vs Females)* | | 0.76 (0.47-1.21) | | - | | 0.98 (0.69-1.40) | | - | |
|  | |  | |  | |  | |  | |
| *Ethnicity* | |  | |  | |  | |  | |
| Black *(vs non-Black)* | | 9.28 (1.42-60.62) | | - | | 3.38 (1.14-10.01) | | - | |
|  | |  | |  | |  | |  | |
| *Highest educational attainment* | |  | |  | |  | |  | |
| Tertiary | | 1.0 | | (0.47-2.14) | | 1.0 | | (0.65-1.54) | |
| Completed high school | | 1.04 (0.42-2.53) | | (0.59-1.81) | | 2.11 (1.21-3.68) | | (1.43-3.13) | |
| Vocational qualifications | | 2.54 (1.09-5.89) | | (1.62-3.98) | | 1.95 (1.15-3.29) | | (1.43-2.66) | |
| Completed lower high school | | 2.10 (0.90-4.91) | | (1.41-3.14) | | 2.39 (1.39-4.11) | | (1.72-3.30) | |
| Completed primary school | | 2.44 (0.87-6.83) | | (1.20-4.98) | | 2.70 (1.53-4.75) | | (1.88-3.88) | |
| No formal education | | 0.67 (0.06-7.86) | | (0.06-6.91) | | 1.86 (0.69-4.99) | | (0.77-4.48) | |
|  | |  | |  | |  | |  | |
| *Baseline income* | |  | |  | |  | |  | |
| High | | 1.0 | | (0.39-2.56) | | 1.0 | | (0.53-1.90) | |
| Medium-high | | 3.09 (1.12-8.52) | | (2.09-4.56) | | 1.52 (0.74-3.12) | | (1.09-2.12) | |
| Medium-low | | 23.16 (8.36-64.16) | | (15.57-34.48) | | 7.16 (3.63-14.13) | | (5.71-8.98) | |
|  | |  | |  | |  | |  | |
| *Number of adult dependants* | |  | |  | |  | |  | |
| Two or more | | 1.0 | | (0.68-1.48) | | 1.0 | | (0.78-1.28) | |
| One | | 1.12 (0.67-1.88) | | (0.79-1.58) | | 2.21 (1.49-3.28) | | (1.63-3.00) | |
| Unrecorded | | 0.97 (0.06-16.63) | | (0.06-16.19) | | 5.55 (0.32-94.89) | | (0.33-93.86) | |
|  | |  | |  | |  | |  | |
| *Number of child dependants* | |  | |  | |  | |  | |
| One or more | | 1.0 | | (0.70-1.44) | | 1.0 | | (0.59-1.70) | |
| None | | 1.71 (1.05-2.77) | | (1.24-2.35) | | 0.90 (0.51-1.61) | | (0.71-1.15) | |
| Unrecorded | | 1.76 (0.37-8.32) | | (0.39-7.97) | | 0.54 (0.20-1.41) | | (0.24-1.20) | |
|  | |  | |  | |  | |  | |
| *Smoking status* | |  | |  | |  | |  | |
| Never smoked | | 1.0 | | (0.71-1.41) | | 1.0 | | (0.79-1.27) | |
| Prior smoker | | 1.16 (0.68-1.98) | | (0.77-1.75) | | 1.22 (0.88-1.69) | | (0.97-1.52) | |
| Current smoker | | 1.76 (0.94-3.30) | | (1.04-2.97) | | 1.29 (0.67-2.51) | | (0.70-2.40) | |
|  | |  | |  | |  | |  | |
| *Prior diseases* | |  | |  | |  | |  | |
| Vascular disease | | 0.74 (0.33-1.63) | | - | | 1.68 (1.04-2.71) | | - | |
| Diabetes mellitus | | 1.73 (0.86-3.46) | | - | | 0.88 (0.58-1.32) | | - | |
|  | |  | |  | |  | |  | |
| *CKD stage* | |  | |  | |  | |  | |
| CKD 3 | | 1.0 | | (0.58-1.71) | | 1.0 | | (0.74-1.35) | |
| CKD 4 | | 1.61 (0.82-3.15) | | (1.08-2.39) | | 1.48 (1.01-2.18) | | (1.17-1.89) | |
| CKD 5 | | 2.79 (1.31-5.95) | | (1.64-4.76) | | 1.23 (0.75-2.01) | | (0.83-1.82) | |
| Dialysis | | 2.16 (1.07-4.36) | | (1.38-3.39) | | 1.91 (1.17-3.11) | | (1.30-2.80) | |

CKD, chronic kidney disease. OR, odds ratio. CI, confidence interval.

*Logistic regression stratified by country

†Data on 4 participants dropped due to colinearity (same country and no fall into poverty).

Wald chi-square test for trend across CKD stage at <60 years, χ^2^=5.95, p=0.0147

Wald chi-square test for trend across CKD stage at ≥60 years, χ^2^=5.01, p=0.0252
