# Supplementary material for: Impact of CKD on Household Income
Source: Kidney Int Rep. 2017 Dec 23;3(3):610–8. doi: 10.1016/j.ekir.2017.12.008 (PMC5976816; doi:10.1016/j.ekir.2017.12.008)
Supplement: Table S8 — Factors associated with the likelihood of a fall into poverty, multivariate logistic regressions following multiple imputation of missing income data. [file mmc8.docx]

**Table S8. Factors associated with the likelihood of a fall into poverty, multivariate logistic regressions following multiple imputation of missing income data**

| **Characteristics at screening** | **OR (Conventional 95% CI)** | **p-value** |
| --- | --- | --- |
| *Age group (years)* |  |  |
| 40-54 | reference |  |
| 55-64 | 1.25 (0.91-1.72) | 0.1675 |
| 65 and older | 1.20 (0.88-1.63) | 0.2422 |
|  |  |  |
| *Sex* |  |  |
| Males *(vs Females)* | 0.90 (0.71-1.15) | 0.4062 |
|  |  |  |
| *Ethnicity* |  |  |
| Black *(vs non-Black)* | 2.86 (1.21-6.76) | 0.0170 |
|  |  |  |
| *Highest educational attainment* |  |  |
| Tertiary | reference |  |
| Completed high school | 1.50 (0.97-2.31) | 0.0666 |
| Vocational qualifications | 1.77 (1.19-2.62) | 0.0047 |
| Completed lower high school | 1.96 (1.29-3.00) | 0.0019 |
| Completed primary school | 2.31 (1.49-3.60) | 0.0002 |
| No formal education | 1.89 (0.84-4.27) | 0.1249 |
| Unrecorded | 0.84 (0.29-2.48) | 0.7517 |
|  |  |  |
| *Baseline income* |  |  |
| High | reference |  |
| Med-high | 2.46 (1.40-4.33) | 0.0018 |
| Med-low | 12.37 (7.19-21.30) | <0.0001 |
|  |  |  |
| *Number of adult dependants* |  |  |
| Two or more | reference |  |
| One | 1.51 (1.15-2.00) | 0.0035 |
| Unrecorded | 0.38 (0.12-1.22) | 0.1024 |
|  |  |  |
| *Number of child dependants* |  |  |
| One or more | reference |  |
| None | 1.13 (0.80-1.59) | 0.4938 |
| Unrecorded | 0.93 (0.48-1.78) | 0.8174 |
|  |  |  |
| *Smoking status* |  |  |
| Never smoked | reference |  |
| Prior smoker | 1.23 (0.95-1.60) | 0.1221 |
| Current smoker | 1.49 (1.01-2.19) | 0.0425 |
|  |  |  |
| *Prior diseases* |  |  |
| Vascular disease | 1.31 (0.90-1.91) | 0.1640 |
| Diabetes mellitus | 1.13 (0.81-1.56) | 0.4683 |
|  |  |  |
| *CKD stage screening* |  |  |
| CKD 3† | reference |  |
| CKD 4 | 1.46 (1.08-1.97) | 0.0139 |
| CKD 5 | 1.40 (0.95-2.08) | 0.0906 |
| Dialysis | 1.73 (1.23-2.43) | 0.0016 |
|  |  |  |
| **Contribution**‡ **of nonfatal vascular events, incident cancers and CKD severity at study end to the likelihood of a fall into poverty** | | |
| *Nonfatal myocardial infarction* | 1.81 (0.91-3.60) | 0.0925 |
| *Nonfatal stroke* | 0.95 (0.45-2.01) | 0.9012 |
| *Incident cancer* | 1.03 (0.70-1.50) | 0.8836 |
| *Composite* nonfatal events* | 1.14 (0.82-1.58) | 0.4453 |
|  |  |  |
| *CKD status at study end* |  |  |
| CKD stage 3-5 | reference |  |
| Transplant^††^ | 0.50 (0.31-0.78) | 0.0027 |
| Dialysis | 1.07 (0.77-1.49) | 0.7009 |

CKD, chronic kidney disease. OR, odds ratio. CI, confidence interval.

Results are based on 20 sets including imputed data with estimation samples between 2776 and 2882 participants. All confidence intervals are compared to the reference category. The logistic regression models were further stratified by country.

†Predominantly CKD stage 3b

‡The categories of post-screening adverse events were introduced one at a time.

*Composite of nonfatal myocardial infarctions, strokes, and incident cancers

Wald chi-square test for trend across CKD stages at screening, χ^2^=8.7, p=0.0033

^††^Transplant at study end split by type of transplant: preemptive transplant OR 0.26 (0.08 -0.91) and non-preemptive transplant OR 0.55 (0.34-0.90) in fully adjusted multivariate model, not significantly different χ^2^=1.21, p=0.2725.
